# TGF-β1/Smad3 Signaling Pathway Mediates T-2 Toxin-Induced Decrease of Type II Collagen in Cultured Rat Chondrocytes

**DOI:** 10.3390/toxins9110359

**Published:** 2017-11-05

**Authors:** Yang Li, Ning Zou, Jing Wang, Ke-Wei Wang, Fu-Yuan Li, Fu-Xun Chen, Bing-Yu Sun, Dian-Jun Sun

**Affiliations:** 1 Center for Endemic Disease Control, Chinese Center for Disease Control and Prevention/Key Lab of Etiology and Epidemiology, Education Bureau of Heilongjiang Province & Ministry of Health (23618504), Harbin Medical University, Harbin 150081, China; yangli9295@hrbmu.edu.cn (Y.L.); zouning@hrbmu.edu.cn (N.Z.); wangjing@hrbmu.edu.cn (J.W.); lifuyuan@hrbmu.edu.cn (F.-Y.L.); sunbingyu@hrbmu.edu.cn (B.-Y.S.); 2 Institute of Cell Biotechnology, China and Russia Medical Research Center, Harbin Medical University, Harbin 150081, China; wangkewei@hrbmu.edu.cn; 3 Gaoqiao Community Health Service Center of Pudong New District, Shanghai 200137, China; gaoqiao-chsc@hotmail.com

**Keywords:** T-2 toxin, chondrocytes, TGF-β1, Smad3, type II collagen

## Abstract

T-2 toxin can cause damage to the articular cartilage, but the molecular mechanism remains unclear. By employing the culture of rat chondrocytes, we investigated the effect of the TGF-β1/Smad3 signaling pathway on the damage to chondrocytes induced by T-2 toxin. It was found that T-2 toxin could reduce cell viability and increased the number of apoptotic cells when compared with the control group. After the addition of the T-2 toxin, the production of type II collagen was reduced at mRNA and protein levels, while the levels of TGF-β1, Smad3, ALK5, and MMP13 were upregulated. The production of the P-Smad3 protein was also increased. Inhibitors of TGF-β1 and Smad3 were able to reverse the effect of the T-2 toxin on the protein level of above-mentioned signaling molecules. The T-2 toxin could promote the level of MMP13 via the stimulation of TGF-β1 signaling in chondrocytes, resulting in the downregulation of type II collagen and chondrocyte damage. Smad3 may be involved in the degradation of type II collagen, but the Smad3 has no connection with the regulation of MMP13 level. This study provides a new clue to elucidate the mechanism of T-2 toxin-induced chondrocyte damage.

## 1. Introduction

T-2 toxin belongs to the family of trichothecenes. The T-2 toxin at a concentration of 2.5 mg/kg can significantly decrease the survival of fish [1]. Previous studies have shown that the T-2 toxin can damage chondrocytes and cartilage tissue [2]. The T-2 toxin can lead to the degradation of type II collagen in chondrocytes [3]. Type II collagen is a main component of the extracellular matrix. T-2 toxin can damage human articular chondrocytes by degradation of the extracellular matrix [3,4]. It has been reported that T-2 toxin caused articular chondrocyte necrosis in experimental animals. The T-2 toxin in contaminated grain can also play an important role in the occurrence of Kashin–Beck disease (KBD) [2,5,6].

Transforming growth factor-β (TGF-β) signaling pathway is widely involved in embryonic development, cell proliferation [7], differentiation, migration, apoptosis, angiogenesis [8], wound repair, immune system regulation [9], bone and joint disease, rheumatic diseases, heart disease, and other diseases [7]. It has been suggested that the TGF-β signaling pathway was mainly mediated by the Smad protein [10]. The Smad proteins are divided into three groups, including receptor-regulated, common and inhibitory types. Smad3 belongs to the receptor-regulated type in the Smad family [11]. ALK5 itself is a TGF-β1 receptor [12]. Some studies examining osteoarthritis in animal models showed that the TGF-β1/Smad3 signaling pathway was implicated in an initiation of cartilage injury via the downregulation of type II collagen [13].

The dynamic distribution of the TGF-β expression had demonstrated that the T-2 toxin could induce an increased expression of TGF-β1 mRNA in epidermal basal cells of mice [14]. A high level of MMP13 could lead to decreased production of type II collagen in rat cartilage [15]. It is deduced that the TGF-β1/Smad3 pathways related to signaling molecules, such as TGF-β1, ALK5, Smad3, type II collagen, and MMP13, are likely to be associated with the T-2 toxin-induced cartilage injury [16].

Until now, the mechanism of T-2 toxin-induced chondrocyte damage has not been fully understood. The relationship between T-2 toxin and TGF-β1/Smad3 pathway-related signaling molecules (i.e., TGF-β1, ALK5, Smad3, type II collagen, and MMP13) has not been entirely elucidated. The present study expounds that the TGF-β1/Smad3 signaling pathway mediates the cartilage injury induced by the T-2 toxin via degradation of type II collagen in rats, which will provide new clues for understanding the mechanism of cartilage injury.

## 2. Results

### 2.1. Effects of T-2 Toxin on Cell Survival Rate of Chondrocytes 

**Figure 1:**
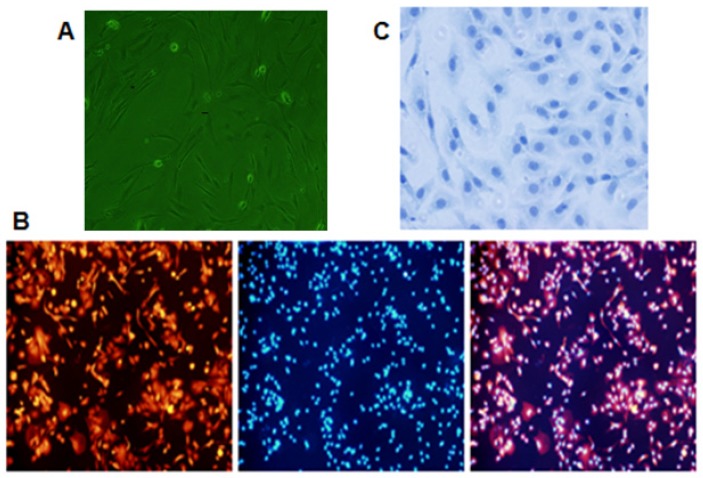
Isolation, culture, and identification of chondrocytes. (**A**) General characteristics of chondrocytes (×200). (**B**) The expression of type II collagen in the third passage chondrocytes (×100). (**C**) The third passage chondrocytes were stained with toluidine blue (×400).

**Figure 2:**
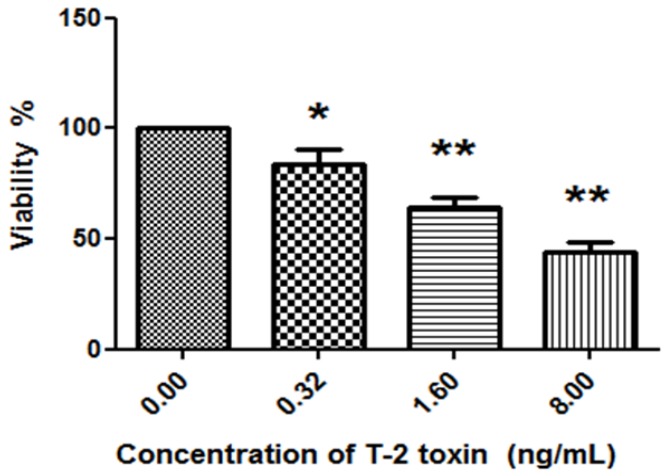
The effect of T-2 toxin on the viability of chondrocytes. Chondrocytes were treated with different concentrations of T-2 toxin (0.00 ng/mL, 0.32 ng/mL, 1.60 ng/mL, 8.00 ng/mL) for 24 h. RTCA-DP (Real Time Cell Analyzer-Dual Plate) system was used to measure the survival rate of chondrocytes. * *p* < 0.05 vs. control, ** *p* < 0.01 vs. control.

Firstly, chondrocytes were primarily cultured and identified as described previously [2,3] (Figure 1). Chondrocytes were treated with different concentrations of T-2 toxin (0.00 ng/mL, 0.32 ng/mL, 1.60 ng/mL, and 8.00 ng/mL) for 24 h. Then, the Real Time Cell Analyzer-Dual Plate (RTCA-DP) system (Roche Applied Science, Indianapolis, IN, USA) was used to measure the survival rate of chondrocytes in each group (Figure 2). The cell viability of chondrocytes was significantly reduced after addition of T-2 toxin when compared with control group. When the concentration of T-2 toxin was increased, the survival rate of chondrocytes was significantly decreased in a dose-dependent manner. Obviously, the addition of T-2 toxin (0.32 ng/mL, 1.60 ng/mL and 8.00 ng/mL) could diminish the cell viability of chondrocytes. 

### 2.2. T-2 Toxin and Ultra-Structure of Chondrocytes

**Figure 3:**
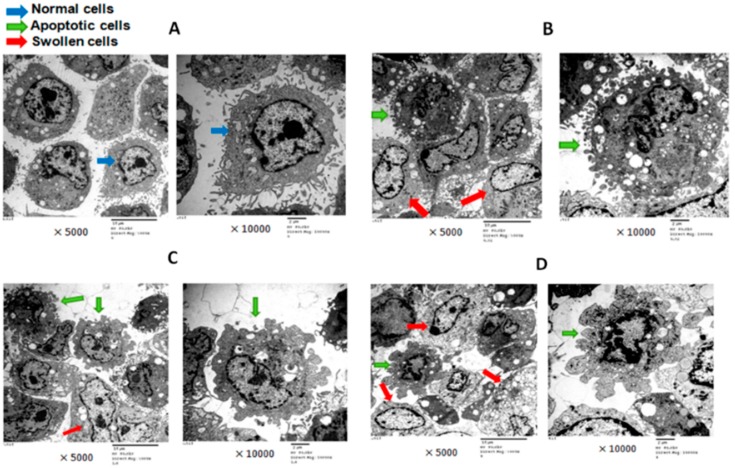
The effect of T-2 toxin on the ultrastructure of chondrocytes. Chondrocytes were treated with different concentrations of T-2 toxin (0.00 ng/mL, 0.32 ng/mL, 1.60 ng/mL, 8.00 ng/mL) for the ultrastructural characteristics of chondrocytes. (**A**) The ultrastructure of chondrocytes in control group. Cells have normal cell structure, including many microvilli on the cell surface, and clear mitochondrial and nucleus structure. (**B**) The ultrastructure of chondrocytes in 0.32 ng/mL T-2 toxin. A few of cells displays swollen, increased intracellular vacuoles, mild pyknosis of cell nucleus. Apoptotic bodies appeared around the cell surface. (**C**) The ultrastructure of chondrocytes in 1.60 ng/mL T-2 toxin. There are some apoptotic cells and swollen cells, accompanied by large autophagosome, nuclei, and mitochondrial swelling. The apoptotic cells indicate transition stage of apoptotic process. (**D**) The ultrastructure of chondrocytes in 8.00 ng/mL of T-2 toxin. There are some apoptotic cells, and the number of swollen cells was further increased. The cell nucleus was condensed and fragmented. * *p* < 0.05 vs. control, ** *p* < 0.01 vs. control.

After treatment with T-2 toxin, the ultrastructure of chondrocytes was observed via transmission electron microscope (TEM). In a normal control group, the visible chondrocytes had irregular shape. There were a lot of microvillus on the cell surface. The nucleus was round or ovoid and located at one side of the cell. The double-layer structure of nuclear membrane was clear and complete. The nuclear pore was visible and obvious. The cytoplasm was rich in rough endoplasmic reticulum in a slightly extended state. The electron density of rough endoplasmic reticulum was uniformly distributed, suggesting that the function of chondrocytes was still in good condition. Scattered mitochondria appeared in the shape of a long kidney-like tube or short rod. The cristae of mitochondria were well-organized. The cell cytoplasm contained abundant free ribosomes, which were evenly dispersed as small clusters (Figure 3A). The addition of T-2 toxin (0.32 ng/mL, 1.60 ng/mL, and 8.00 ng/mL) resulted in decreased organelles in the cytoplasm, nuclear chromatin plaques, karyopycnosis, and the nuclear membrane thickened. In this state, the double membrane structure became unclear and blurred. The microvilli of the chondrocytes were lost gradually. After an increased concentration of T-2 toxin, the number of rough endoplasmic reticulum decreased, and their cavities were dilated. Vacuole degeneration and medullary change in mitochondria occurred. The cellular structure was abnormal, and many chondrocytes died of apoptosis. Apoptotic body appeared around the cell membrane. Cell swelling was accumulated. Increased vacuoles and mitochondrial electron density were observed. Cell necrosis could be also found. It was worth noting that the effect of T-2 toxin on the ultrastructural changes of chondrocytes were aggravated (Figure 3B–D).

### 2.3. Effects of T-2 Toxin on Collagen Degradation-Related Proteins

**Figure 4:**
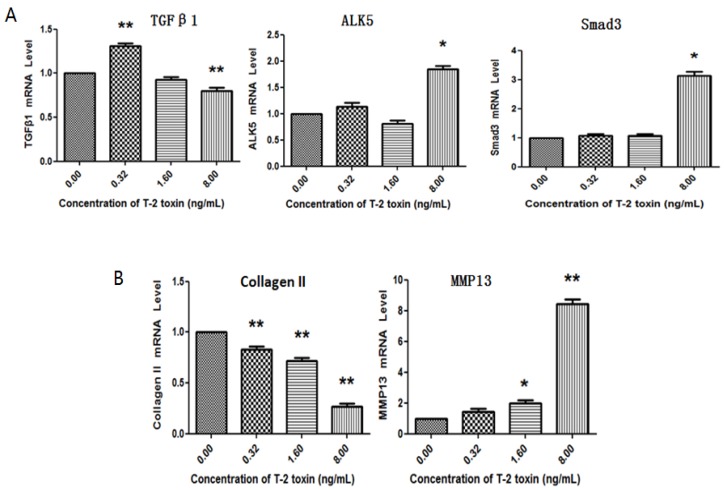
T-2 toxin induced mRNA changes of collagen degradation-related TGF-β1, ALK5, Smad3, collagen II, and MMP13. The expression of TGF-β1, ALK5, Smad3, collagen II, and MMP13 was analyzed by RT-PCR (reverse transcription-polymerase chain reaction) at the level of mRNA, subsequent to treatment with different concentrations of T-2 toxin (0.00 ng/mL, 0.32 ng/mL, 1.60 ng/mL, 8.00 ng/mL). * p < 0.05 vs. control, ** p < 0.01 vs. control.

**Figure 5:**
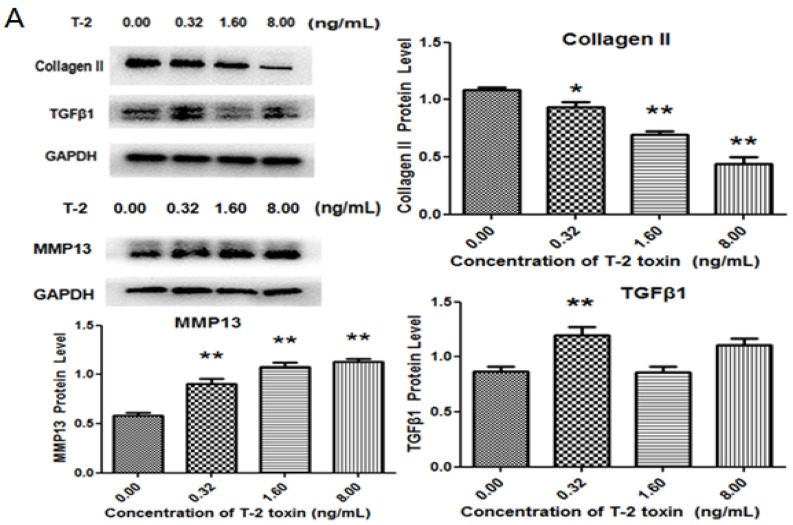

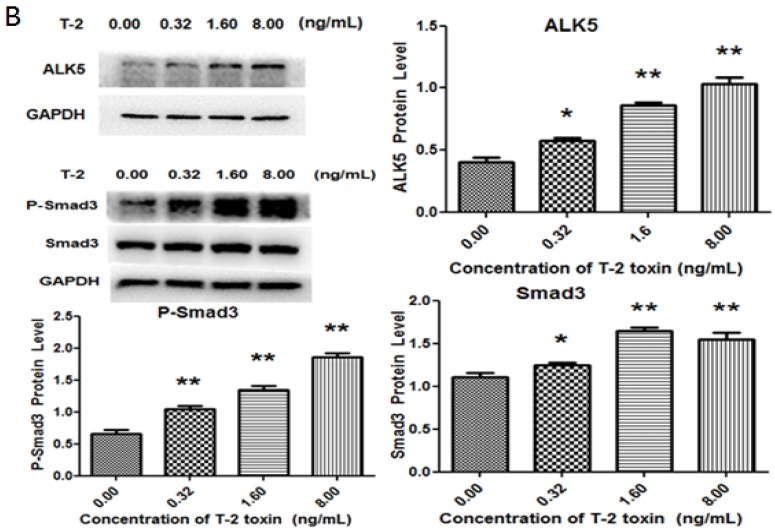
T-2 toxin induced changes of collagen degradation-related proteins. Western blot was used to detect the protein production of (**A**) TGF-β1, MMP13, type II collagen, (**B**) ALK5, P-Smad3, and Smad3 subsequent to treatment with different concentrations of T-2 toxin (0.00 ng/mL, 0.32 ng/mL, 1.60 ng/mL, 8.00 ng/mL). * p < 0.05 vs. control, ** p < 0.01 vs. control.

To investigate the mechanism of T-2 toxin-induced damage, we examined the changes in collagen degradation-related proteins. Chondrocytes were treated with or without T-2 toxin for 24 h, before RT-PCR was used to measure the level of mRNAs. When compared with the control, we have found that TGF-β1 was upregulated after treatment with 0.32 ng/mL of T-2 toxin, while T-2 toxin at a concentration of 1.60 ng/mL had no significant effect on TGF-β1 production. Furthermore, 8.00 ng/mL of T-2 toxin was able to inhibit the level of TGF-β1. Although 0.32 ng/mL and 1.60 ng/mL of T-2 toxin did not affect the expression of ALK5, a high concentration (8.00 ng/mL) of T-2 toxin could upregulate the level of ALK5. The level of Smad3 was increased in chondrocytes after treatment with 8.00 ng/mL T-2 toxin, while low concentration (0.32 ng/mL and 1.60 ng/mL) did not affect the level of Smad3 mRNA. The T-2 toxin decreased the level of mRNA of type II collagen in a dose-dependent manner. The T-2 toxin at concentration of 1.60 ng/mL and 8.00 ng/mL increased the expression of MMP13, but 0.32 ng/mL of T-2 toxin had no effect on the expression (Figure 4). The protein levels of MMP13, ALK5, Smad3, and P-Smad3 were significantly increased, the production of type II collagen was reduced in a dose-dependent manner in T-2 toxin-treated cells, TGF-β1 showed a trend level similar to its mRNA when compared with the control (Figure 5). 

### 2.4. TGF-β1 Inhibitor and Smad3 Inhibitor Blocked the Effect of T-2 Toxin on Chondrocytes

**Figure 6:**
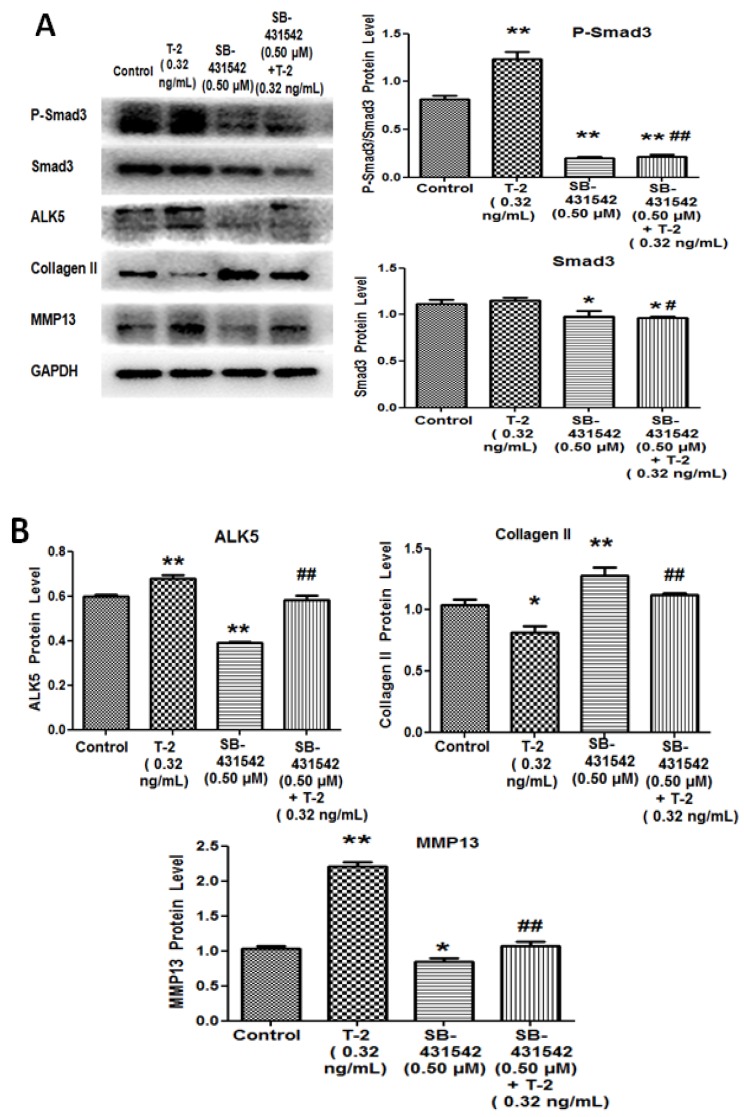

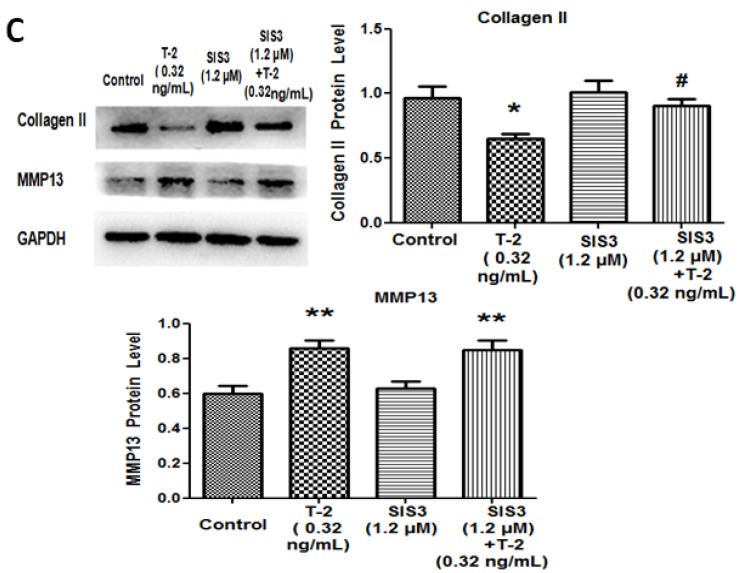
TGF-β1 and Smad3 inhibitors could block the effect of T-2 toxin. After incubation with (**A**,**B**)SB-431542 (0.50 μM of TGF-β1 inhibitor) or (**C**) SIS3 (1.2 μM of Smad3 inhibitor) for 1 h, chondrocytes were treated with T-2 toxin for another 24 h. Western blot was used to detect protein levels of P-Smad3, Smad3, ALK5, type II collagen, and MMP13 in chondrocytes. * p < 0.05 vs. control, ** p < 0.01 vs. control, *^#^ p* < 0.05 vs. T-2 (0.32 ng/mL), *^##^ p* < 0.01 vs. T-2 (0.32 ng/mL).

It has been reported that TGF-β1 was involved in the occurrence and development of osteoarthrosis [17], and we further investigated whether the TGF-β1 signaling pathway affected the effect of T-2 toxin on chondrocytes. In this study, we found that 0.32 ng/mL of T-2 toxin can induce a significant increase in TGF-β1 at both mRNA and protein levels. Thus, 0.32 ng/mL of T-2 toxin was used for the subsequent mechanism-related study. In the next experiment, we used SB-431542 0.50 μM as the TGF-β1 inhibitor, and SIS3 1.20 μM as the Smad3 inhibitor. As described in Figure 6A,B, compared with the control group, 0.32 ng/mL of T-2 toxin increased the production of P-Smad3, ALK5 and MMP13, but decreased the level of type II collagen. However, the TGF-β1 inhibitor (SB-431542) had an opposite effect on the production of P-Smad3, ALK5, MMP13, and type II collagen, and decreased the level of Smad3. We pretreated chondrocytes with TGF-β1 inhibitor, before treating cells with 0.32 ng/mL of T-2 toxin for 24 h. Compared with the group of T-2 toxin, the TGF-β1 inhibitor attenuated levels of P-Smad3, Smad3, ALK5, and MMP13, which were promoted by the T-2 toxin. The TGF-β1 inhibitor also increased the production of type II collagen, which were decreased by the T-2 toxin (Figure 6A,B). On the other hand, when the chondrocytes were pretreated with the Smad3 inhibitor (SIS3) for one hour, and then incubated with 0.32 ng/mL of T-2 toxin for 24 h, the Smad3 inhibitor increased the down-regulation of type II collagen that was reduced due to the treatment of T-2 toxin. However, the level of MMP13 could be not altered subsequent to the administration of Smad3 inhibitor (Figure 6C), which suggested that changes to MMP13 did not occur through activation of Smad3. But, the Smad3 was important for the degradation of T-2 toxin-induced type II collagen. These data demonstrate that the TGF-β1/Smad3 signaling pathway mediates T-2 toxin-induced damage of chondrocytes.

## 3. Discussion

The cartilage tissue is composed of chondrocytes, extracellular matrix and collagen fibers. The articular hyaline cartilage is enriched with type II collagen, which is secreted by the chondrocytes. Thus, chondrocyte damage is an initial process of cartilage injury, including KBD occurrence. It has been reported that T-2 toxin could induce apoptosis of chondrocytes, which may cause or worsen the development of the KBD [18,19]. However, the mechanism of T-2 toxin-induced injury in chondrocytes remains not to be determined. In this study, the culture of chondrocytes was utilized to investigate the toxic role of T-2 toxin. After identification of chondrocytes using immunochemistry of type II collagen, fluorescence-labeling and toluidine blue staining techniques, we found that the isolated cells from articular cartilage were almost 100% chondrocytes. Their further culture would be eligible for studying the characteristics of chondrocytes and the toxicology of T-2 toxin.

It has been reported that the T-2 toxin caused degenerative articular changes similar to spontaneous osteoarthritis in rats [20]. The T-2 toxin inhibited proliferation and promoted apoptosis in chondrocytes, which may be associated with the reactive oxygen species (ROS)-mediated mitochondrial pathway [21]. To investigate the effect of T-2 toxin on chondrocytes, RTCA-DP was performed. The survival rate of chondrocytes was gradually decreased with an increased concentration of T-2 toxin, which indicated that T-2 toxin inhibited the proliferation of chondrocytes in a dose-dependent manner. After ultrastructural changes of chondrocytes were detected by TEM, results showed that the number of swollen and apoptotic chondrocytes increased, and the damage of chondrocytes were aggravated after the addition of T-2 toxin. Those data indicate that T-2 toxin damages chondrocytes. There was a dose-dependent relationship between the concentration of T-2 toxin and the damage of chondrocytes.

It has been reported that the TGF-β1/Smad3 signaling pathway was essential for the inhibition of chondrocyte differentiation [21,22]. Chondrocytes will undergo abnormal terminal differentiation, ultimately leading to osteoarthritis without these inhibitive signals [2]. In this study, we found that the levels of TGF-β1/Smad3 signaling pathway-related factors (TGF-β1, ALK5, Smad3, type II collagen, and MMP13) were significantly altered in T-2 toxin-treated chondrocytes at both mRNA and protein levels when compared with control group. Type II collagen produced by chondrocytes is a major component of articular cartilage. While the concentration of T-2 toxin was increased, the production of type II collagen was dramatically reduced [23]. 

At the same time, the mRNA and protein levels of MMP13 were significantly elevated in this study [24]. Studies have shown that the degradation of components (mainly composed of collagen) was increased in patients with osteoarthritis, while MMP13 involved in the degradation of collagen was significantly increased. This suggests that the loss of extracellular matrix is due to the enhanced collagen-degrading enzyme (MMP13) activity. MMP13 is the key enzyme to result in the loss of collagen. The balance between synthesis and decomposition of collagen II could be disturbed by MMP13, which caused the damage of chondrocytes [3]. These data indicated that T-2 toxin could induce the degradation of extracellular matrix in chondrocytes. The degradation of type II collagen caused by T-2 toxin in chondrocytes was achieved by improving the production of MMP13.

Levels of ALK5, Smad3, type II collagen, and MMP13 located downstream of the TGF-β1/Smad3 signaling pathway were further analyzed by Western blot, following treatment with TGF-β1 inhibitor and 0.32 ng/mL of T-2 toxin in chondrocytes. The results showed that the production of those proteins in all groups had a remarkable change, especially type II collagen. When compared with the control group, the level of type II collagen in T-2 toxin-treated cells decreased markedly, but in the group treated by TGF-β1 inhibitor and T-2 toxin, the expression of type II collagen did not change significantly. However, TGF-β1 inhibitor could increase the production of type II collagen, which was reduced by T-2 toxin. And the TGF-β1 inhibitor attenuated the T-2 toxin-induced increase in MMP13. This indicates that the T-2 toxin promotes the production of MMP13 and decreases the level of type II collagen. The T-2 toxin has a toxic effect on chondrocytes, probably through the TGF-β1-related signaling pathway. 

Smad2 and Smad3 also play an important role in TGF-β signaling pathway. According to the previous studies, gene mutations of Smad3 could be associated with the pathogenesis of human osteoarthritis. Moreover, the effect of Smad3 was stronger than the effect of Smad2 [25,26], so we only detected the level of Smad3 in the present study. Smad3 plays an important role in TGF-β1 signal transduction [11], so Smad3 may be involved in T-2 toxin-induced degradation of type II collagen in chondrocytes. Our study showed that the Smad3 inhibitor could increase the expression of type II collagen, which was reduced by T-2 toxin, although there was no connection between the expression of Smad3 and MMP13. This indicates that T-2 toxin could reduce the expression of type II collagen and damage chondrocytes through the activation of Smad3, but the effect of MMP13 on chondrocytes is not mediated through regulation of Smad3.

In summary, T-2 toxin promoted the production of MMP13, and increased the degradation of type II collagen in chondrocytes through the TGF-β1/Smad3 signaling pathway, which ultimately led to damaged chondrocytes. However, T-2 toxin stimulated the expression of MMP13, but not through Smad3. Moreover, Smad3-induced reduction of type II collagen was not mediated through the up-regulation of MMP13. Therefore, some other factors may exist, which could interact with Smad3. The exact mechanism of the chondrocyte damage remains unclear, so further efforts are still needed in the future. 

## 4. Materials and Methods

All the experiments were approved by Institutional Research Board of Harbin Medical University (No.: HMUIRB20170024; Date: 11 July 2017).

### 4.1. Cell Culture

The neonatal SD (Sprague Dawley) rats (age of 1–3 days) were purchased from the Experimental Animal Center of the Affiliated Second Hospital of Harbin Medical University (Harbin, China). After the animals were sacrificed, they were disinfected with 75% ethanol, before their limbs were separated under a sterile environment. Cartilage tissues were cut into tiny pieces, which were digested with 0.25% trypsin at 37 °C for 20 min, and then centrifuged at 1000 rpm for 5 min. Subsequently, supernatants were removed, and the tissues were digested with 2 g/L of type II collagenase at 37 °C for 5 h. A serum-containing medium was used to terminate the digestion. Cells were filtered with a stainless steel mesh in aseptic condition. After centrifugation at 1000 rpm for 5 min, the cells were resuspended (the cell number is about 1 × 10^6^ of each rat) in RPMl 1640 supplemented with 10% BI (Biological Industries) fetal calf serum, 100 U/mL of penicillin G, and 100 μg/mL of streptomycin. The suspended cells were incubated with 5% CO_2_ at 37 °C. After having been plated overnight, chondrocytes attached to the wall of the culture flask. The stretched cells exhibited irregular spindle or polygonal shapes. The cultured chondrocytes from neonatal SD rats contained abundant cytoplasm and had a clear nucleus (Figure 1A). Overall, the cultured cells maintained the general characteristics of chondrocytes that were similar to those in vivo. All experiments were performed using the third generation of chondrocytes. The subsequent experiments were performed at least three independent times. 

### 4.2. Immunofluorescence

The third generation of chondrocytes was seeded at concentration of 0.16 × 10^6^ cells in a 6-well plate with preset sterilized coverslips. After the cells adhered, they were washed with PBS and fixed with 4% paraformaldehyde for 30 min. After having been washed with PBS three times, the cells were blocked with 5% BSA at 37 °C for 30 min, before being incubated with primary antibodies (Rabbit Anti-COL2A1/Type II collagen, BOSTER, Pleasanton, CA, USA) at 4 °C overnight. The cells were incubated with secondary antibodies (Rhodamine-Conjugated AffiniPure Goat Anti-Rabbit IgG, purchased from ZSGB-BIO, Beijing, China) at 37 °C for 60 min, before the cells were counterstained with 4′, 6-diamidino-2-phenylindole (DAPI) for 3–5 min. Immunofluorescence was observed using the fluorescence microscope. As the cartilage matrix is enriched with type II collagen, the synthesis and secretion of the type II collagen can be used as a specific maker for identifying the differentiation phenotype of chondrocytes [16]. Under the fluorescence microscope, the type II collagen secreted by the cultured chondrocytes was dyed in red, and the nucleus was stained in blue (Figure 1B).

### 4.3. Toluidine Blue Staining

The chondrocytes were further identified with toluidine blue dye. The third generation of chondrocytes at a concentration of 0.16 × 10^6^ cells was cultured in a 6-well plate with preset coverslips. The cells were washed with PBS, fixed with 4% paraformaldehyde for 20 minutes, and subsequently fixed with 75% ethanol for 20 min, respectively. Thereafter, 1% toluidine blue was added, and then samples were put in a 60 °C oven for 2–3 h. The cells were rinsed with anhydrous ethanol quickly, air-dried, then sealed in a coverslip using a neutral gum. Finally, these stained cells were observed under a microscope. The cytoplasm was dyed in light blue, while the nuclei were stained in dark blue. The majority of cells were mononuclear (Figure 1C).

### 4.4. Cell Viability Assay

The third generation of chondrocytes were seeded in a sterile 16-well plate (approximately 1 × 10^4^ cells per well). The cultured cells were adherent in 24 hours. They were randomly divided into four groups and treated with T-2 toxin (Sigma-Aldrich, St. Louis, MO, USA) at different concentrations, including 0.00 ng/mL, 0.32 ng/mL, 1.60 ng/mL, and 8.00 ng/mL. Cell viability was measured by RTCA-DP.

### 4.5. Observation of Chondrocytes by Transmission Electron Microscopy

The chondrocytes were seeded in a culture flask and allowed to grow into the third generation (approximately 1 × 10^6^ cells per culture bottle). The chondrocytes from each group were collected in a 1.5 mL eppendorf tube and fixed with 2.5% glutaraldehyde after the administration of T-2 toxin for 24 h. The ultrastructural characteristics of chondrocytes were observed by transmission electron microscopy.

### 4.6. Real Time PCR

Total RNA was extracted with the TRizol kit. cDNA was synthesized under the following conditions: 25 °C for 10 min, 50 °C for 1 min, 85 °C for 5 min, and 4 °C for 1 h. The 7500 fast PCR system was used to detect the transcription levels of mRNA. The primer sequences used are listed in Table 1.

### 4.7. Western Blot

A total of 100 μL of lysate was added into an EP tube containing chondrocytes. The specimen was put on ice for vortex oscillation. Cell lysates were centrifuged for 5 min at 14,000 rpm, and at 4 °C. The supernatant was collected. The concentration of protein was determined by the BCA kit according to the manufacturer’s instruction. The denatured specimen was run on SDS-PAGE gels, and the separated proteins were transferred to polyvinylidene difluoride membranes. The membranes were blocked with 5% BSA for 30 min, before they were incubated by primary antibodies (Table 2) at 4 °C overnight. The secondary antibody (peroxidase-conjugated AffiniPure goat anti-rabbit IgG) was added at room temperature for 1 h. 

### 4.8. Statistical Analysis

All of the data were represented as mean ± standard deviation. SPSS software version 17.0 was used to analyze differences among the groups. *p* < 0.05 was considered as significant. *p* < 0.01 was considered as highly significant.

## Figures and Tables

**Table 1:** Primer sequence.

No.	mRNA	Primer Sequence (5′-3′)
1	TGFβ1	F:GGAAGGACCTGGGTTGGAAG
		R:GTAGTAGACGATGGGCAGTGG
2	ALK5	F:AGGATGACTTACAGAGGCTTAGAC
		R:TGATACCCAGTGACTCAAGGAAG
3	Smad3	F:AGTTCTCCAGAGTTAAAAGCGA
		R:CTTGACCGCCTTCTCGCA
4	Type II collagen	F:GCCAGGATGCCCGAAAATTAG
		R:GTCACCTCTGGGTCCTTGTTC
5	MMP13	F:TGCATACGAGCATCCATCCC
		R:CTCAAAGTGAACCGCAGCAC
6	GAPDH	F:AACTCCCATTCTTCCACCTTTG
		R:CTCTTGCTCTCAGTATCCTTGC

**Table 2:** Dilution ratio of antibody in Western blot.

No.	Antibody	MW (kDa)	Dilution Ratio	Sources (Company)
1	TGF-β1	44	1:500	Proteintech
2	ALK5	56	1:1000	abcam
3	P-Smad3	52	1:1000	Cell Signaling TECHNOLOGY
4	Smad3	48	1:3000	abcam
5	Type II collagen	142	1:10,000	abcam
6	MMP13	54	1:3000	abcam
7	GAPDH	37	1:1000	GOOD HERE
8	Peroxidase-conjugated Afinipure goat anti-rabbit IgG	0.8 mg/mL	1:25,000	ZSGB-BIO

## References

[1] Manning B.B., Li M.H. (2003). Response of channel catfish to diets containing T-2 toxin. J. Aquat. Anim. Health.

[2] Guan F., Li S., Wang Z.L., Yang H., Xue S., Wang W., Song D., Zhou X., Zhou W., Chen J.H. (2013). Histopathology of chondronecrosis development in knee articular cartilage in a rat model of Kashin-Beck disease using T-2 toxin and selenium deficiency conditions. Rheumatol. Int..

[3] Chen J.H., Cao J.L., Chu Y.L., Yang Z.T., Shi Z.L., Wang H.L., Guo X., Wang Z.L. (2006). Protective effect of selenium against T-2 toxin-induced inhibition of chondrocyte aggrecan and collagen II synthesis. J. South. Med. Univ..

[4] Chen J., Chu Y., Cao J., Wang W., Liu J., Wang J. (2011). Effects of T-2 toxin and selenium on chondrocyte expression of matrix metalloproteinases (MMP-1, MMP-13), α2-macroglobulin (α2M) and TIMPs. Toxicol. In Vitro.

[5] Lu M., Cao J., Liu F., Li S., Chen J., Fu Q., Zhang Z., Liu J., Luo M., Wang J. (2012). The effects of mycotoxins and selenium deficiency on tissue-engineered cartilage. Cells Tissues Organs.

[6] Chen J., Luo M., Wang W., Zhang Z., He Y., Duance V.C., Hughes C.E., Caterson B., Cao J. (2015). Altered proteolytic activity and expression of MMPs and aggrecanases and their inhibitors in Kashin-Beck disease. J. Orthop. Res..

[7] Bruna A., Darken R.S., Rojo F., Ocaña A., Peñuelas S., Arias A., Paris R., Tortosa A., Mora J., Baselga J. (2007). High TGFbeta-Smad activity confers poor prognosis in glioma patients and promotes cell proliferation depending on the methylation of the PDGF-B gene. Cancer Cell.

[8] Guo P., Hu B., Gu W., Xu L., Wang D., Huang H.J., Cavenee W.K., Cheng S.Y. (2003). Platelet-derived growth factor-B enhances glioma angiogenesis by stimulating vascular endothelial growth factor expression in tumor endothelia and by promoting pericyte recruitment. Am. J. Pathol..

[9] Guo X., Zuo H., Cao C.X., Zhang Y., Geng D., Zhang Z.T., Zhang Y.G., von der Mark K., von der Mark H. (2006). Abnormal expression of Col X, PTHrP, TGF-β, bFGF, and VEGF in cartilage with Kashin-Beck disease. J. Bone Miner. Metab..

[10] Shi Y., Massagué J. (2003). Mechanisms of TGF-beta signaling from cell membrane to the nucleus. Cell.

[11] Macias M.J., Martin-Malpartida P., Massagué J. (2015). Structural determinants of Smad function in TGF-β signaling. Trends Biochem. Sci..

[12] Ma B., Zhou P.Y., Ni W., Wei W., Ben D.F., Lu W., Xia Z.F. (2013). Inhibition of activin receptor-like kinase 5 induces matrix metallopeptidase 9 expression and aggravates lipopolysaccharide-induced pulmonary injury in mice. Eur. Rev. Med. Pharmacol. Sci..

[13] Aref-Eshghi E., Liu M., Harper P.E., Doré J., Martin G., Furey A., Green R., Rahman P., Zhai G. (2015). Overexpression of MMP13 in human osteoarthritic cartilage is associated with the SMAD-independent TGF-β signalling pathway. Arthritis Res. Ther..

[14] Albarenque S.M., Shinozuka J., Suzuki K., Nakayama H., Doi K. (2000). Kinetics and distribution of transforming growth factor (TGF)-beta 1 mRNA in the dorsal skin of hypotrichotic WBN/ILA-Ht rats following topical application of T-2 toxin. Exp. Toxicol. Pathol..

[15] Wang J., Ma J., Gu J.H., Wang F.Y., Shang X.S., Tao H.R., Wang X. (2017). Regulation of type II collagen, matrix metalloproteinase-13 and cell proliferation by interleukin-1β is mediated by curcumin via inhibition of NF-κB signaling in rat chondrocytes. Mol. Med. Rep..

[16] Pullig O., Kladny B., Weseloh G., Swoboda B. (1999). Metabolic activation of chondrocytes in human osteoarthritis. Expression of type II collagen. Z. Orthop. Ihre Grenzgeb..

[17] Wang M., Shen J., Jin H., Im H.J., Sandy J., Chen D. (2011). Recent progress in understanding molecular mechanisms of cartilage degeneration during osteoarthritis. Ann. N. Y. Acad. Sci..

[18] Chen J., Chu Y., Cao J., Yang Z., Guo X., Wang Z. (2006). T-2 toxin induces apoptosis, and selenium partly blocks, T-2 toxin induced apoptosis in chondrocytes through modulation of the Bax/Bcl-2 ratio. Food Chem. Toxicol..

[19] Wang S.J., Guo X., Zuo H., Zhang Y.G., Xu P., Ping Z.G., Zhang Z.T., Geng D. (2006). Chondrocytc apoptosis and expression of Bcl-2, Bax, Fas, and iNOS in articular cartilage in patients with Kaschin-Beck disease. J. Rheumatol..

[20] Wang L.H., Fu Y., Shi Y.X., Wang W.G. (2011). T-2 toxin induces degenerative articular changes in rodents: Link to Kaschin-Beck disease. Toxicol. Pathol..

[21] Serra R., Johnson M., Filvaroff E.H., LaBorde J., Sheehan D.M., Derynck R., Moses H.L. (1997). Expression of a truncated, kinase-defective TGF-beta type II receptor in mouse skeletal tissue promotes terminal chondrocyte differentiation and osteoarthritis. J. Cell Biol..

[22] Yang X., Letterio J.J., Lechleider R.J., Chen L., Hayman R., Gu H., Roberts A.B., Deng C. (1999). Targeted disruption of SMAD3 results in impaired mucosal immunity and diminished T cell responsiveness to TGF-β. EMBO J..

[23] Wang X., Zhang Y., Chang Y., Duan D., Sun Z., Guo X. (2016). Elevation of IGFBP2 contributes to mycotoxin T-2-induced chondrocyte injury and metabolism. Biochem. Biophys. Res. Commun..

[24] Yan D., Kang P., Yang J., Shen B., Zhou Z., Duan L., Deng J., Huang H., Pei F.X. (2010). The effect of Kashin-Beck disease-affected feed and T-2 toxin on the bone development of Wistar rats. Int. J. Rheum. Dis..

[25] Yao J.-Y., Wang Y., An J., Mao C.-M., Hou N., Lv Y.-X., Wang Y.-L., Cui F., Huang M., Yang X. (2003). Mutation analysis of the *Smad3* gene in human osteoarthritis. Eur. J. Hum. Genet..

[26] Alvarez J., Serra R. (2004). Unique and redundant roles of Smad3 in TGF-beta-mediated regulation of long bone development in organ culture. Dev. Dyn..

